# The quality of father-child feeding interactions mediates the effect of maternal depression on children’s psychopathological symptoms

**DOI:** 10.3389/fpsyt.2022.968171

**Published:** 2022-08-22

**Authors:** Silvia Cimino, Renata Tambelli, Paola Di Vito, Gessica D’Angeli, Luca Cerniglia

**Affiliations:** ^1^Department of Dynamic, Clinical and Health Psychology, Sapienza University of Rome, Rome, Italy; ^2^International Telematic University Uninettuno, Rome, Italy

**Keywords:** postnatal depression, parent-infant interaction, fathers, children’s emotional/behavioral functioning, child developmental

## Abstract

Research has shown that Postnatal maternal depression (PND) is associated with children’s emotional and behavioral problems during infancy, but the possible effect of father-child relationship quality on this association is yet to be thoroughly investigated. We recruited 401 families (802 parents; 401 children) *via* mental health clinics in Central Italy. We divided families into two groups: Group 1 included families with mothers with PND; Group 2 included families with mothers without PND (control group). The assessment took place at T1 (18 months of age of children) and T2 (36 months of age of children): postnatal maternal depression was measured through the Edinburgh Postnatal Depression Scale (EPDS); parent-child relationship quality was assessed through the Scale for the Assessment of Feeding Interactions (SVIA); and the child emotional–behavioral functioning was evaluated with the Child-Behavior-Checklist (CBCL). Compared to the control group, the children of the groups where mothers had PND, showed overall higher scores (i.e., more maladaptive) on the CBCL. A direct effect of postnatal maternal depression on children’s emotional-behavioral functioning was found, both at T1 and at T2. A mediation effect of father-child relationship quality between postnatal maternal depression and child outcomes was also found. These results could inform prevention and intervention programs in families with mothers with PND.

## Introduction

Postnatal depression (PND) affects 10–22% of all mothers (1, 2). Epidemiological studies suggest that postnatal depression symptoms tends to spontaneously remit within 6 months (3). However, some residual depressive symptoms are common up to 1 year after delivery (4, 5). PND is known to be associated with adverse and negative outcomes both for mothers and their infants, and the pathways by which these adverse consequences can occur may be disparate (6). The Developmental Psychopathology framework (7, 8) has highlighted consistent empirical evidence on the association between the quality of the caregiving system and protective and risk factors in childhood psychopathology. According to this perspective, negative outcomes may be considered as referring to the individual emotional well-being of the parent and/or of the children, and to the reduced quality of their interactions (9). PND can affect the health and development of a new-born, as well as mother-infant relationship. Research suggests that PND has a direct impact on a mother’s ability to respond sensitively to her infant, affecting the quality of mother-infant relationship (10, 11). PND has an effect not only on mothers’ psychological well-being and mother-infant relationship, but also on the child’s development (12–14). Some studies have found that PND can influence the quality of parenting (15–17), in particular impairing parenting practices related to feeding and breastfeeding (18, 19). The interaction between mothers diagnosed with depression and their children is often severely impaired (20, 21), especially for what concerns feeding. What seems to be particularly impaired is the continuity of emotional interaction due to increased withdrawal and loss of initiative or intrusive overstimulation. Depression, in fact, can damage maternal caregiving capacity and, consequently, also the ability of the mother-child dyad, to reciprocally regulate their interaction (22). Lack of reciprocal regulation can affect social exchanges and affect regulation, leading to poor interactions characterized by negative emotions (23). On the other hand, seminal literature (24, 25) has suggested that receptive and positive parenting in the first year of life, characterized by face-to-face contact, positive facial expression, is of crucial importance for children’s psychological development and can be considered a protective factor against negative psychopathological outcomes in children. Specifically, as for child outcomes, the most common ones include cognitive and emotional-behavioral problems (26). PND not only may have a direct negative impact on the infants because of their exposure to their mothers’ disorder, but it has been suggested to have an indirect effect *via* the impact of the parental disorder on interpersonal behavior (e.g., interactions, parenting, feeding) (4).

Although researchers have dedicated increasing attention to fathers, the role they play in the infant well-being and emotional development in families where the mother is diagnosed with PND has yet to be thoroughly considered. Previous studies suggested that fathers can mediate the degree of risk these children experience being exposed to maternal depression (27). Moreover, other studies noted that children’s maladaptive interactions with their depressed mothers were not extended to their fathers who were not depressed (28, 29). Children with both their caregiver with depression show higher risk of a negative outcome when compared to those with only one depressed parent (30). An interesting study by Vakrat (29) suggests that good fathering can reduce this risk by half. Coherently with the Developmental Psychopathology theoretical approach, psychopathological outcomes in children are mediated by individual, relational, genetic, and environmental protective and/or risk factors (31). The quality of parent-infant interactions plays an important role in shaping children’s development (32). Though the role of maternal PND on children’s emotional-behavioral functioning has been widely investigated (33–35), few studies have specifically used observational tools in order to investigate the quality of interactive patterns during feeding in families of children in their first 3 years of life where mothers were diagnosed with PND (36). Both quantitative and qualitative research has suggested that mothers with postnatal depression symptoms experienced difficulties during feeding interactions (37). Often, interactive exchanges during meals between mothers with postnatal depression and their children can be characterized by reduced face-to-face interactions, reduced frequency of positive facial expression, asynchronies in turn-taking (38). The adaptive development of self-regulatory skills in the infant is promoted by sensitive and responsive care. Some authors suggested that the quality of caregiver- infant interactions can vary over time, especially in the first 3 years of a child’s life. In fact, a bulk of literature (39, 40) suggests using longitudinal study in order to investigate the quality of parent-child interactions as they may be quickly subject to rapid changes in the first years of life. Moreover, maternal depression is known to be as the most prevalent risk impacting the parent’s ability to provide good-enough caregiving, particularly when this symptomatology occurs during the child’s first years of life (41).

In brief, previous literature posits that if the mother suffers from PND, this can affect the regulatory capacity of the mother-child interaction (22); and this can lead to poor interactions resulting in the expression of maladaptive emotions and behaviors. Good-enough interaction is crucial for the child’s psychological development (23). However, some gaps in literature have not been filled, such as the reason why some children living in an environment where the mother suffers from psychopathological problems such as depression, do not develop psychopathological or behavioral symptoms. Therefore, we set ourselves the goal of understanding if (and if so, how) the quality of both the mother’s relationship with the child and the father’s relationship with the child affects the child’s emotional and behavioral behavior. This study proposed two different assessment points (T1 when the child was 18 months old and T2 when the child was 36 months old) that correspond to specific developmental touch points. At around 18 months of age, children generally develop a more complex interaction and are able to follow the caregiver with their gaze, for example during playtime or mealtime and around 36 months of age children begin to interact with adults communicating experiences or ideas (42). In this study, we recruited a sample of families where mothers showed clinical symptoms of postnatal depression (Group 1) and we compared it to a sample of mothers without symptoms, matched by the same demographic characteristics (Group 2). Our aims were to assess maternal perception of their offspring’s emotional-behavioral functioning and verify possible differences between the two groups; assess mother–child’s and father–child’s quality of the feeding interactions in the two groups at T1 and T2, verifying possible significant differences; and investigate the possible role of the quality of father–infant interactions during feeding in mediating between mother’s PND and offspring’s emotional-behavioral difficulties. Based on previous premises, we expected that: (1) Children of mothers in Group 1 showed more emotional-behavioral problems when compared with children of mothers in Group 2; (2) Mother-child and father-child interaction were characterized by maladaptive exchanges in Group 1 rather than Group 2; (3) Fathers had a mediating role between mothers’ PND and offspring’s emotional-behavioral difficulties—that is, positive father-child interactions could have a protective effect on children’s mental health.

## Materials and methods

### Participants and procedure

We only recruited families when they were responsible for their children nutrition and were both primary caregivers. We excluded from the study participants that referred medical diagnosis of the child, medication or drug consumption (parents or child), or parents that were currently undergoing a psychiatric or treatment. The research was approved by the Ethical Committee of the Psychology Faculty at Sapienza, University of Rome before its start, in accordance with the Declaration of Helsinki. We recruited a total of 401 families (*N* = 802 parents; *N* = 401 children). We evaluated mothers’ and fathers’ interaction with their children with the Italian translation (43) of the Feeding Scale (44). We divided our sample into two groups based on EPDS cut-off scores. In Group 1 (*N* = 227 families) mothers exceeded the clinical cut-off, while Group 2 (*N* = 173 families) did not and was our control. In this study 57.1% of mothers exceeded the clinical cut-off of the Edinburgh Postnatal Depression Scale (EPDS). In Group 1, subjects (depressed mothers) were recruited through agreements with child mental health services and hospitals located in the area, while in Group 2, subjects were recruited from preschools located in the area. Data were collected at two assessment points: at T1 when the children were 18 months old and at T2 when the children were 36 (T2) months old. Mother-child and father-child feeding interactions were video-recorded during a meal at home. The parent-child exchanges were coded with the Scala di Valutazione Interazioni Alimentari (43, 44). Both at T1 and T2, mothers were asked to complete the Child Behavior Checklist questionnaire (45), in order to assess their child’s emotional behavioral functioning. The team taking part in the research was composed by psychologist and clinicians that were specifically trained in the use of the tools applied in this study. Table 1 describes demographic characteristics of the sample. All subjects provided provide data for the second visit at 36 months. All families were Caucasian. The majority of mothers having completed high school or university (89%); 96% of mothers were married, and all households were of average socioeconomic status (94% had an annual income of 25,000–30,000 Euros).

**TABLE 1:** Demographic characteristics of our sample.

	Group 1	Group 2
		
*N*	227	174
Children’s gender	116 (50.7%) M 111 (49.3%) F	87 (50%) M 87 (50%) F
Children’s age (months) T1	19,53 (2,41)	19,56 (2,76)
Children’s age (month) T2	34,29 (1,25)	34,29 (1,52)
Mothers’ age (years)	32,32 (2,7)	33,03 (3,7)
Father’s age (years)	35,48 (4,9)	35,65 (4,5)

### Tools

The Feeding interactions Assessment Scale (SVIA) was used to assess mother-child and father-child interactions during mealtime. The instrument was administered separately to mother-child and father-child dyads during a main meal in their home. Parents also completed the Child Behavior Checklist 11/2-5 (CBCL) (EPDS), described below, at T1, T2. The mother’s depression was assessed *via* the Edinburgh Postnatal Depression Scale (EPDS), described below, at T1 and T2.

#### Edinburgh postnatal depression scale

The EPDS is a self-report questionnaire to screen depression symptoms after childbirth. It consists of 10 items with four options different for each item, rated from 0 to 3 (Yes, most of the time; Not very often; No, not at all), assessing the woman’s mood (e.g., I have blamed myself unnecessarily when things went wrong; I have felt sad or miserable). The total possible score ranges from 0 to 30. A higher score indicates a higher level of depression symptoms, and the recent individual patient data meta-analysis established 11 as a cut-off score (46). Two Italian studies have validated this instrument for the Italian population, showing acceptable psychometric properties (47, 48).

#### Scale for the assessment of feeding interactions

For this study, the Italian version of the Feeding Scale (44) for the evaluation of mother-infant feeding interaction was used (43). The scale measures a wide range of interactive behaviors and identifies normal and/or risky relational feeding dynamics between mother and child and the Feeding Scale is applied to children from 0 to 36 months (43). Parent–infant interactions during feeding are recorded for at least 20 min, and then a wide range of interactive mother–infant behaviors are coded and evaluated. The Scale created for the Italian version has 41 items, representing four subscales: (1) Parent’s affective states; (2) Interactive conflict; (3) Food refusal behavior; and (4) Dyad’s affective state. The scores, ranging from 0 to 3 (none, a little, quite, very much) are measured on a 4-point Likert scale. The SVIA, in terms of internal consistency, shows good reliability (Cronbach’s α, 0.79–0.96). In this study, we used the cut-off of 55 for relationship quality in general. When the scores are greater than or equal to 55 the quality of the parent-child relationship is considered low-functional or dysfunctional whereas when the score is less than 54 the parent-child relationship is considered functional.

#### Child behavior check-list

The CBCL is a questionnaire filled out by parents or caregivers which can detect the child’s abilities and a wide range of behavioral and emotional problems in young children that may require clinical attention. The CBCL 1½–5 (45) is composed of 99 items. This tool also includes open qualitative questions in order to obtain additional information from the parent that spends more time with the child. The caregiver is asked to rate their child’s behavior for how true each item is at the present time or in the last 6 months (0 = not true; 1 = somewhat or sometimes true; 2 = very true or often true). For example: afraid to try new things; clings to adults or too dependent. For the purpose of our study, in line with (49), we used the 2 summary scales of the CBCL. The Internalizing Problems Scale consists of four syndrome subscales: Emotionally Reactive, Anxious/Depressed, Somatic Complaints, and Withdrawn. The Externalizing Problems Scale is composed of two syndrome subscales: Attention Problems and Aggressive Behavior. The CBCL 1½–5 has high test–retest reliability and high internal consistency (45). In the present study, we used the Italian validated versions and the Italian cut-off values (50).

### Data analysis

Data were analyzed using IBM SPSS statistics (Version 27) and PROCESS MACRO for Windows (51). Descriptive statistics were used to determine the reliability of the measures, mean scores, and percentages. During the preliminary analysis, Cronbach’s α coefficient was used to assess reliability of the instruments. For the quantitative analysis, mixed analyses of variance (ANOVAs) were conducted on the data in the CBCL scales at T1 and at T2. A regression model was used to determine the effect of maternal post-natal depression at T1 on children’s emotional-behavioral functioning at T2 and the possible mediating role of fathers.

## Results

### Child’s internalizing and externalizing symptoms

Table 2 reports mean scores and standard deviations for the CBCL syndromic scales. Children in Group 1 showed higher scores on all the scales, both at T1 and T2 than children in Group 2. However, none of the children exceeded the clinical cut-offs for Externalizing and Internalizing scores of the CBCL in both groups. The scores did not significantly change from T1 to T2 in both groups. In order to investigate the presence of differences between our two groups in the perceived child’s emotional-behavioral symptomatology, an ANOVA was conducted on these data. Group was considered as between subject factor (1 vs. 2) and Time (T1 vs. T2) as within subject factor, considering Externalizing and Internalizing symptoms as dependent variables. Results showed a significant Group effect (Wilk’s Lambda = 0.170, *F* = 481.7, *p* = 0.000) both on Internalizing (*F* = 31.08, *p* = 0.000) and Externalizing symptoms (*F* = 37.84, *p* = 0.000), while Time was not significant. Children whose mothers showed clinical scores on the EPDS seemed to exhibit a higher degree–while below the cut-off–of externalizing and internalizing problems but these problems did not seem to increase or decrease over time.

**TABLE 2:** Average scores and standard deviations of the Child Behavior Cheklist (CBCL) internalizing and externalizing scales.

		Group 1	Group 2
			
		*M* (SD)	*M* (SD)
Internalizing	T1	26.84 (10.78)	20.34 (13.04)
	T2	24.10 (9.87)	20.68 (13.43)
Externalizing	T1	16.76 (7.48)	13.35 (7.8)
	T2	17.46 (7.74)	12.53 (8.58)
Total	T1	141.55 (33.29)	85.43 (39.75)
	T2	137.47 (22.31)	74.82 (48.35)

### Mother–child feeding interactions: Differences between groups

Table 3 shows mean scores and standard deviations of the SVIA subscales for mother–child feeding interactions. In order to investigate the presence of differences between the two groups in the quality of mother-child feeding interactions, a mixed ANOVA was conducted on these data. Group was the between-subject factor (1 vs. 2) and Time was the within-subject factor (T1 vs. T2). We considered each SVIA subscale during mother-child interaction as dependent variable. Results showed a significant Group effect (Wilk’s Lambda = 0.10, *F* = 879.71, *p* = 0.000). Univariate tests reported a statistically significant effect on all the SVIA subscales: Maternal Affective State (*F* = 290.11, *p* = 0.000), Interactive Conflict (*F* = 309.73, *p* = 0.000), Food Refusal Behavior (*F* = 295.78 *p* = 0.000), Dyad’s Affective State (*F* = 351.87, *p* = 0.000). Mothers who showed clinical scores on the EPDS seemed to exhibit worse feeding interaction patterns than mothers whose EPDS scores were below the cut-off. Coherently with the fact that no intervention was administered, the effect of Time was not significant, meaning that the scores in both groups remained substantially stable over time.

**TABLE 3:** Average scores and standard deviations of the Scala di Valutazione dell’Interazione Alimentare (SVIA) subscales applied during mother-child and father-child feeding interactions.

		Mothers group 1	Mothers group 2	Fathers group 1	Fathers group 2
					
		*M* (SD)	*M* (SD)	*M* (SD)	*M* (SD)
Affective state	T1	19.73 (6.03)	10.45 (4.33)	18.04 (7.29)	13.72 (6.69)
	T2	18.66 (5.68)	5.87 (4.08)	13.58 (8.20)	14.51 (8.39)
Interactive conflict	T1	18.22 (5.64)	9.19 (4.14)	16.34 (6.38)	11.74 (6.37)
	T2	17.16 (4.45)	5.96 (4.36)	12.60 (7.18)	13.58 (7.11)
Food refusal behavior	T1	10.71 (3.46)	5.51 (2.18)	9.41 (3.91)	7.32 (3.42)
	T2	9.90 (2.86)	3.20 (2.18)	7.33 (4.49)	7.94(4.56)
Dyad’s affective state	T1	11.82 (4.12)	5.09 (2.57)	10.68 (4.32)	6.93 (4.28)
	T2	10.75 (3.36)	3.27 (2.27)	7.96 (4.76)	8.30 (4.75)
SVIA total	T1	60.49 (18.68)	30.26 (12.47)	54.47 (21.11)	39.73 (20.35)
	T2	56.49 (15.49)	18.32 (12.35)	41.49 (24.22)	44.34 (24.39)

### Father–child feeding interactions: Differences between groups

Table 3 shows mean scores and standard deviations of the SVIA subscales for father–child feeding interactions. In order to investigate the presence of differences between the two groups in the quality of father-child feeding interactions, a mixed ANOVA was conducted on these data. Group was the between-subject factor (1 vs. 2) and Time was the within-subject factor (T1 vs. T2). We considered each SVIA subscale during mother-child interaction as a dependent variable. Results showed a significant Group effect (Wilk’s Lambda = 0.15, *F* = 544.97, *p* = 0.000). Univariate tests reported a statistically significant effect on all the SVIA subscales: Paternal Affective State (*F* = 38.39, *p* = 0.000), Interactive Conflict (*F* = 53.01, *p* = 0.000), Food Refusal Behavior (*F* = 33.84, *p* = 0.000), Dyad’s Affective State (*F* = 76.82, *p* = 0.000). Fathers whose partners showed clinical scores on the EPDS seemed to exhibit worse feeding interaction patterns than fathers whose partners’ EPDS scores were below the cut-off. Coherently with the fact that no intervention was administered, the effect of Time was not significant, meaning that the scores in both groups remained substantially stable over time.

### The predictive effect of maternal depression on children’s emotional behavioral functioning

In Group 1 maternal depression seemed to predict the following CBCL subscales: Emotionally Reactive, Somatic Complaints, Attention Problems, and Aggressive behavior as well as the Externalizing symptoms summary scale. In Group 2, EPDS scores seemed to have an effect predicting the Total CBCL scores and summary scales (Externalizing and Internalizing symptoms) but did not have a specific influence on a specific area of the child’s emotional-behavioral functioning. It seemed that mother’s depression in Group 1 influences a specific externalizing area of children’s emotional behavioral functioning as other studies have previously posited.

### The predictive effect of father-child relationship on children’s internalizing and externalizing symptoms at T1 and T2

In order to investigate the direct predictive effect of father-child relationship on children’s emotional behavioral functioning, we run two separate regressions per Group per assessment point. In Group 1, father-child relationship was not significant in predicting internalizing symptoms at T1. However, father-child interactions during feeding seem to significantly predict externalizing symptoms at T1 (R2 = 0.715, *F* = 104.97, *p* = 0.000). Specifically, Interactive Conflict (β = 0.77, *p* = 0.01), Food Refusal (β = 1.56, *p* = 0.000), Dyad’s Affective State (β = 1.41, *p* = 0.000). Affective State was not significant. However, at T2, father-child relationship significantly predicted both internalizing (R2 = 0.43, *F* = 31.66, *p* = 0.000) and externalizing symptoms (R2 = 0.42, *F* = 31.36, *p* = 0.000). Specifically, for what concerns Internalizing symptoms, Food Refusal (β = 0.61, *p* = 0.02), Dyad’s Affective State (β = 1.14 *p* = 0.001). Affective State and Interactive conflict were not significant. For what concerns Externalizing symptoms, Interactive Conflict (β = 0.86, *p* = 0.02), Food Refusal (β = 0.67, *p* = 0.01), Dyad’s Affective State (β = 1.12, *p* = 0.001). Affective state was not significant. In Group 2, father- child relationship was not significant in predicting internalizing or externalizing symptoms at T1. At T2, father-child relationship significantly predicted internalizing symptoms (R2 = 0.22, *F* = 16.10, *p* = 0.000). Only Interactive Conflict was significant (β = 0.53, *p* = 0.04). Father-child relationship was not significant in predicting externalizing symptoms at T2.

### Investigating the mediating role of parent-child relationship quality between maternal depression and child’s symptoms

We used Andrew Hayes’ Process (v.3.3) macro that allowed us to test a possible mediation effect on a dependent variable when the dependent variable is binary. We wanted to investigate the possible effect of mothers’ depressive symptoms on the child’s emotional behavioral functioning with a possible mediating effect of the quality of father-child relation. The direct effect path from mother’s depression to child’s symptoms was positive and statistically significant (R = 0.623, s.e. = 1.9437, *p* < 0.000) indicating that mothers with higher scores on depression are more likely to have children that exceed the cut-off for the CBCL total scores than those scoring lower on depression. The direct effect of mother’s depression on father-child relation is negative but not significant. The direct effect of father-child relation and mother-child relation on child’s symptoms is significant, indicating that fathers and mothers scoring lower on the SVIA are less likely to have children that exceed the cut-off for the CBCL total score. The indirect effect is tested using non-parametric bootstrapping. In our case the indirect effect (IE = 1.0827) is statistically significant (Boot SE = 0.1951; Boot LLCI = 0.7144; Boot ULCI = 1.4749) suggesting that fathers’ and mothers’ lower SVIA scores can help mediate mothers’ effect on children’s symptoms.

## Discussion

Based on previous literature discusses above we expected that children of mothers with clinical depressive symptoms showed higher emotional-behavioral problems when compared with children of mothers without symptoms. We also anticipated that mother-child and father-child interactions during feeding were characterized by higher maladaptive exchanges in Group 1 than Group 2. Moreover, we estimated that fathers had a mediating role between mothers’ PND and offspring’s emotional-behavioral difficulties—that is, positive father-child interactions could have a protective effect on children’s mental health.

Our first hypothesis was confirmed. Consistently with other studies (52, 53), children belonging to Group 1 reported significantly higher levels of internalizing and externalizing emotional-behavioral difficulties. However, contrary to other studies (54, 55), while children of mothers who showed clinical depressive symptoms presented more severe symptoms than offspring in Group 2, these problems did not increase or decrease from 18 to 36 months of age. Moreover, our data showed that the quality of mother-child feeding interactions was associated with offspring symptoms, in the sense that poorer quality of the exchanges was linked to higher psychopathological risk in offspring.

Our hypothesis was confirmed also for our second aim. In fact, our results showed that Group 1 had significantly higher scores on all SVIA subscales, meaning that both mothers and fathers presented more maladaptive feeding interactive exchanges when mothers with depression were present in the family compared to Group 2. We speculate that mothers with postnatal depressive symptoms and their partners experienced the moment of feeding as particularly difficult; both dyads (mother-child and father-child) were characterized by maladaptive patterns of interactive exchanges such as asynchronized turn-taking, lack of responsiveness and sensitivity, and the general emotional climate is negative. Our results were in line with previous literature that underlined how mother’s depression can negatively affect the child’s behavioral and emotional functioning and the general emotional climate of the family (56–58). It has been posited that the negative outcomes on offspring of depressed mothers are associated with mothers’ difficulty in assuming a parental role (59). However, our data confirmed that notwithstanding the direct negative effects of maternal psychopathology on their child’s behavior and affects, as demonstrated in consolidated literature (6, 60), this impact should be considered as the result of maladaptive dyadic interactions (61, 62).

To tap our third aim and consistently with the Developmental Psychopathology framework (32), we created a predictive model to investigate the specific role of the quality of parent-child interactions on their offspring’s emotional/behavioral functioning. Our findings confirm our hypothesis of a direct influence of maternal postnatal depression on the psychological well-being of the child. Moreover, our model confirmed that father-child interactions during feeding mediate the influence of maternal depressive symptoms on offspring’s problems. This result is particularly relevant. Notwithstanding the fact that depressed mothers often have impaired interactive exchanges with their offspring [scarce face-to-face contact, negative affect, lack of responsiveness, passivity, or intrusiveness; (63)], our findings suggest that while the direct effect of postnatal depression is strong in predicting negative outcomes in children, the quality of paternal interactions during routine activities, which include affective exchanges, is of crucial importance in fostering children’s well-being (64, 65). This is in line with other empirical evidence of mediating role of parent behavior in child maladjustment (40, 64, 66, 67). Fathers, according to Lamb, connect with their children in a special and unique way that differs from the way women interact with their children. Physical contact and rough-and-tumble play appear to be more common in fathers’ relationship patterns with their children. Feeding interactions between fathers and their children have been described as different from mothers-children’s ones in that fathers seem more focused on making the child finish the meal impeding offspring distractions (68). These characteristics appear to have a special function in supporting the child’s adaptive emotional-regulation processes, which eventually lead to less psychopathological symptoms in offspring. Importantly, such processes start to mature in the first 3 years of life. For these reasons, it appears critical to include fathers in studies that aim to evaluate the quality of parent-infant interactions in young children; while a growing number of scientific papers are examining fathers’ role in children’s developmental and psychological outcomes, literature describing the use of observational tools with daily routines (e.g., play and/or feeding) is still scarce. The present study aimed at filling this gap.

## Limitation and future direction

The present study has some limitations. We used parent report-form questionnaires to assess children’s emotional-behavioral functioning. More objective measures are needed to minimize the risk of parents’ distorted perception of offspring’s psychological functioning (69, 70). Moreover, we didn’t evaluate fathers’ psychopathological risk; however, we included in the study only fathers who did not report any psychiatric diagnosis when recruiting our sample. Apart from the above limitations, our study could add to the previous literature and inform clinical practice. First, the results of the study confirmed and emphasized the importance of early identification of a possible eating disorder in at-risk children. Thanks to the clinical-diagnostic assessment of feeding interactions and monitoring the quality of relational patterns it is possible to analyse the connections between various maternal psychopathologies and children’s symptoms, to formulate preventions and interventions plans (71). Moreover, the quality of parent-child relationship was assessed through an observational method, administered by trained mental health clinicians. This method assesses behavioral and emotional patterns which include individual characteristics of the child and of mother but also dyadic characteristics. Due to changes in the family’s organization, which now includes shared responsibilities between mothers and fathers in the rearing of children (e.g., in feeding and sleeping routines), with fathers involved in the care of their sons and daughters as much as mothers, who are now more often employed outside the home, the fathers’ role has been addressed as a protective and/or adjunct risk factor for the onset of psychological difficulties in children.

Notwithstanding the limitation of our work, this study addresses a research field that is still under-explored. Investigating the psychological and interactional functioning of fathers of children with depressed mothers could help understanding the links and the relationship between caregivers’ psychological aspects and children’s outcomes. This could be useful, as suggested by other literature (72), to include the role of the father–which research is showing to be of crucial importance–when evaluating the possibility of prevention and intervention programs in families with children with depressed mothers.

## Data availability statement

The raw data supporting the conclusions of this article will be made available by the authors, without undue reservation.

## Ethics statement

The studies involving human participants were reviewed and approved by Ethical Committee of the Psychology Faculty at Sapienza, University of Rome. Written informed consent to participate in this study was provided by the participants’ legal guardian/next of kin.

## Author contributions

SC and LC contributed to conception and design of the study. GD’A organized the database. PD performed the statistical analysis. PD and LC wrote the first draft of the manuscript. PD, GD’A, LC, and SC wrote sections of the manuscript. All authors contributed to the article and approved the submitted version.
